# Life Satisfaction Index among Elderly People Residing in Gorgan and Its Correlation with Certain Demographic Factors in 2013

**DOI:** 10.5539/gjhs.v8n8p41

**Published:** 2015-12-17

**Authors:** Maryam Chehregosha, Amir Bastaminia, Fatemeh Vahidian, Azam Mohammadi, Aliakbar Aghaeinejad, Ensiyeh Jamshidi, Afsaneh Ghasemi

**Affiliations:** 1 MS in Nursing, Faculty Member of the Paramedicine School, Golestan University of Medical Sciences, Iran; 2 PhD Student of Gerontology, University of Social Welfare and Rehabilitation Sciences, Tehran, Iran; 3 PhD Student in Geography and Urban Planning, Department of Geography, School of Humanities, University of Yazd, Yazd, Iran; 4 BSc of Laboratory Science, Students Research Committee, Golestan University of Medical Sciences, Gorgan, Iran; 5 BSc of Nursing, Golestan University of Medical Sciences, Gorgan, Iran; 6 MSc in Nursing, Faculty Member of the Paramedicine School, Golestan University of Medical Sciences, Gorgan, Iran; 7 PhD Candidate in Health Education and Promotion, Community Based Participatory Research Center, Iranian Institute for Reduction of High-Risk Behaviors, Tehran University of Medical Sciences, Tehran, Iran; 8 Assistant Professor of Health Education and Promotion, Department of Public Health, School of Public Health, Fasa University of Medical Sciences, Fasa, Iran

**Keywords:** perception of aging, the elderly, demographic factors, LSIA

## Abstract

**Background:**

Aging is a universal phenomenon that will present itself as a dominant social and welfare challenge.

**Aim:**

This study was to examine life satisfaction among people residing in Gorgan and its correlation with certain demographic factors in 2013.

**Methods:**

A total of 250 elder people were selected for the study through the convenience sampling during 4 months. Data collected through life satisfaction index-A (LSIA). This instrument consists of 5 subscales, including, zest for life, resolution and fortitude, congruence between desired and achieved goals, positive self-concept and mood tone. The Multiple Linear Regression analysis was used in order to determine factors influencing the overall LSIA.

**Results:**

The overall LSIA score was 22.1 ± 7.5 with the maximum and minimum mean scores pertaining to the resolution and fortitude (6.1 ± 2.5) and the positive self-concept (3.1 ± 1.2) subscales, respectively. Level of education, type of living and gender were variables influencing the overall LSIA (P<0.05).

**Conclusion:**

Given the overall LSIA, it appears that future plans for this age group should be seriously revised along with cultural plans for promoting reverence for old age in the general public.

## 1. Background

Aging is a universal phenomenon that will present itself as a dominant social and welfare challenge posed to developing countries in the near future (Organization, 2012). Since the mid-twentieth century the percentage of elderly people in the world’s population has been steadily increasing. This trend is more evident in developing countries. There are 554 million elderly people living in developing nations-a fivefold increase since 1950. The number of elderly people in these regions will further triple by 2050 to total 1.6 billion people. Although the speed of population change in developed countries is significant, it is lower than that of developed countries (Nations, 2001).

According to the national 2012 census of population and housing, over 8.2% of Iran’s population is composed elderly people over the age of 60. This rate is expected to reach one-third of the population by the year 2050 (Iran, 2012). Based on the statistics provided, it can be argued that Iran is currently transitioning from being a country with a young population to a country with a middle-aged population and will soon join other countries with a predominantly older population (Sharifzadeh, Moodi, & Akhbari, 2010).

An aging population leads to new opportunities: People live long, healthier lives and are able to work for more years (Bloom, Boersch-Supan, McGee, & Seike, 2011). Dynamic aging is the goal, meaning, as the elderly population increases, the quality of their life should also be underlined. Dynamic aging is the proactive process of optimizing opportunities for attaining health, active engagement and safety so as to improve the quality of life for elderly people (Rezvani, Mansourian, Ahmadadadi, & Parvai Here-Dasht, 2013).

However, these changes also bring about challenges for health care services. When people live longer, they require more attention to their health, and therefore are often hospitalized for chronic and degenerative diseases, functional dependency, mental health and cognitive disorders. As a consequence, health service providers are faced with increasing costs in order to maintain their services (Dahlan, Nicol, & Maciver, 2010).

Because of their decreased functional abilities and heightened vulnerability, seniors require significant care and support. The needs of seniors should be assessed in physical, social and psychological aspects (Babapour, Raheb, & Eglima, 2014).

Life satisfaction denotes the individual’s positive attitude toward the world in which he lives (Koohbanani, Dastjerdi, Vahidi, & Far, 2013). Satisfaction and need have a positive correlation. They both change over time and are subject to numerous factors in society (Krause & Hayward, 2013). Incongruence between goals, desires and needs, which often occurs due to particular issues and problems, causes dissatisfaction (Masoudi et al., 2010). Life satisfaction is not a persistent objective quality; rather, it is susceptible to contextual changes and is judged based on peoples own perception and interpretation (Aishvarya et al., 2014).

Subjective quality of life can be defined by using terms such as “life satisfaction,” “subjective well-being” and “happiness.” Life satisfaction includes factors such as health, education, interpersonal communication and socioeconomic status which are applicable in assessing the conditions of overall life (Beyaztas, Kurt, & Bolayir, 2012).

Therefore, the attempt to increase human life span should be further accompanied by an emphasis on quality of life, meaningfulness and well-being. Life satisfaction is therefore an important concept that needs careful consideration (Noroozian, 2012).

In Iran, several studies have been conducted to study life satisfaction in older adults. Some studies have used the Diener Life Satisfaction Scale (Babapour et al., 2014; Hojjati, Sharifnia, Hassanalipour, Akhonzadeh, & Asayesh, 2011; Jamalzadeh & Golzary, 2014; Mobasheri et al., 2014). Only one study has applied a life satisfaction questionnaire (LSI [Emami, Molavi, & Kalantary, 2014]), which is typically not used to assess the factors associated with life satisfaction. The present study was thus conducted in order to examine the LSIA among the elderly population of Gorgan and its correlation with certain demographic factors.

## 2. Method

### 2.1 Study Design

A total of 250 elderly (age 60 or over) individuals residing in Gorgan were selected for the present cross-sectional study in 2013 using the convenience sampling method. Data were collected through face-to-face interviews. As no fixed site existed for easy access to middle-aged and elderly people, the researcher had to visit Jahandidegan Charity Institution for the Elderly, local parks and the municipal retirement center of Gorgan over a period of 4 months in order to interview the target population. All participants were over the age of 50 and were capable of visual and verbal communication in the form of responding to questions and did not suffer from any psychological problems or other conditions disturbing their consciousness during the initial examination. In order for the individuals to understand the questions precisely and avoid misunderstanding the concept of the questions, the researcher attended in the aforementioned locations and asked the elderly individuals all of the questions of the questionnaire verbally, even if the elderly were literate, and able to read and write.

### 2.2 Data Collection Instruments

The instruments used for collecting data include a demographic and clinical characteristics section and the Life Satisfaction Index-A (LSIA). In the demographic and clinical characteristics section information such as age, gender, education and lifestyle was taken. In order to understand whether a participant was currently suffering from a chronic disease, the researcher asked the participants what types of chronic diseases they experienced, if any. He asked the elderly participants if they had any history of cardiovascular disease, renal failure, cancer, diabetes, hypercholesterolemia, hypertension, respiratory diseases, Eye Diseases and Bone Joint and Muscle Disorders. It is worth noting that the data was obtained based on the oral statements of the patient, and no documents were gathered to verify their responses. The life satisfaction criteria among elderly people were first exploited by Neugarten et al., in 1961 (Neugarten, Havighurst, & Tobin, 1961). This instrument consists of 5 subscales, including zest for life (4 items), resolution and fortitude (5 items), congruence between desired and achieved goals (3 items), positive self-concept (3 items) and mood tone (5 items). Respondents expressed their agreement or disagreement with the statements based on a 3-point Likert scale (agree=2 point; disagree=1 point; and I don’t know=0 points). The range of scores was between 0 and 40. In order to measure the score for each subscale, the scores obtained for all the statements classified under that subscale should be added together and divided by the number of statements. Niknami et al., (Niknami M, Namjou A, Baghaei M, & Atrkar Roshan Z, 2010) used the Persian version of this instrument and reported its reliability to be 0.7.

### 2.3 Statistical Analysis

In order to attain objectives of the study, normality of data was first examined using the Kolmogorov - Smirnov test. Descriptive statistical indicators were used in the study including the mean and standard deviation for the mean score of overall life satisfaction and its dimensions.

The t-test compared the average life satisfaction score by gender (male/female) and the presence of chronic disease (yes/no). Furthermore, the comparison of the average life satisfaction scores by age (60-75, 75-90 and over 90 years), educational status (illiterate, elementary, secondary, high school and college and advanced degrees), and living status (with family, with wife/husband, single) was analyzed by the ANOVA test.

Ultimately, the Multiple Linear Regression analysis was used to assess the effect of the independent variables on overall LSIA so that the effect of the confounding factors would be eliminated. It should be noted that the regression model analyzed the age variable as a quantity, gender (referent: male) and the presence/absence of a chronic disease (referent: absence) as binary variables, and the level of education (referent: illiterate) and living arrangements (referent: with family) as dummy variables. Data were analyzed in SPSS 20 software and the level of significance was set at 0.05.

## 3. Results

The mean age of participants was 67.6 (SD=6.8), ranging from 60 to 92 years old. The majority of participants were male (54 %), illiterate (59.6%) and living with their family (55.2%) in terms of their living arrangement.

Generally 75.6% of participants reported chronic conditions with the highest percentage related to hypertension (24.1 %) and diabetes (14.9%) respectively.

Investigating the frequency of the LSIA did not judge 40% of the participants (100 individuals) to be satisfied, 26% (65 people) satisfied nor dissatisfied and 34% (85 people) dissatisfied. Table 1 presents the specifications of participants in detail.

**Table 1:** Association of respondents’ characteristics and total score of LSIA by using ANOVA and Independent Sample t-test

Variable		Frequency	Percent	LSIA Mean (SD)	P-value
Age	(60- 75) (Year)	211	84.4	22.5 (7.5)	0.11†
	(75- 90) (Year)	35	14	19.7 (6.8)	
	(< 90 (Year)	4	1.6	20.3 (4.6)	
Gender	Men	135	54	22.9 (8)	0.06††
	Women	115	46	21.1 (6.7)	
Educational Level	Illiterate	149	59.6	21.9 (7.4)	0.16†
	Elementary	65	26	21.3 (7.6)	
	Secondary school	8	3.2	24.2 (5.1)	
	High school	17	6.8	22.8 (8.2)	
	University	11	4.4	27 (6.3)	
History of Chronic	No	61	24.4	19.5 (7.1)	0.002*††
	Yes	189	75.6	22.9 (7.4)	
Living status	With family	138	55.2	21.3 (7.3)	0.18†
	With husband/wife	92	36.8	22.9 (7.5)	
	Single	20	8	23.6 (8.1)	

† One-way ANOVA

†† Independent Sample t- test

* Significant at α level less than 0.05.

In terms of the dimensions of the LSIA, the highest and lowest mean scores pertained to the resolution and fortitude (6.1 ± 2.5) and the positive self-concept (3.1 ± 1.2) subscales, in respective order (Table 2).

**Table 2:** Distribution of mean and standard deviation of life satisfaction and its dimensions in the study population (n = 250)

Variable	Mean	SD	Minimum	Maximum
Total score of Life Satisfaction	22.1	7.5	12	37
Zest for life	4.9	1.9	1	8
Resolution and fortitude	6.1	2.5	2	10
Congruence between desired and Achieved goals	4.1	1.2	1	6
Positive self-concept	3.1	1.2	1	6
Mood tone	4	2	2	8

The Multiple Linear Regression model was used to determine factors influencing overall LSIA. Table 3 presents variables that have a significant correlation with LSIA. Chronic disease (<0.001, CI: 2.6 - 6.7), Type of life with husband (0.02, CI: 0.4 - 4.4), Educational level: Middle school (0.03, CI: 0.6 - 11.2), Educational level: University (0.04, CI: 0.2 - 9.4) were meaning full statistical significant correlation with LSIA.

**Table 3:** The association between respondents’ characteristics and total score of LSIA by using Multiple Linear Regression

	95% Confidence Interval			
Variables	β	Lower	Upper	P-value
Constant	22.4	12.3	32.519	<0.001
Gender	-1.6	-3.5	0.4	0.1
Chronic disease	4.5	2.6	6.7	<0.001^*^
Age	-0.07	-0.2	0.1	0.4
Life status: With husband/wife	2.4	0.4	4.4	0.02^*^
Life status: Single	3.1	-0.5	6.6	0.1
Educational level: Secondary school	5.9	0.6	11.2	0.03^*^
Educational level: High school	0.06	-3.6	3.7	0.9
Educational level: University	4.8	0.2	9.4	0.04^*^

* Significant at α level less than 0.05.

## 4. Discussion

In this study, 40 percent of the elderly participants were satisfied with their lives. In a study by Li et al., in 2014, a group of elderly individuals age 60 and over was interviewed and 54.6 percent of the participants living in urban areas were satisfied with their lives (Li et al., 2014). In the research study of Won and Choi in 2013, 25 elder people were assessed, and 85.5 percent of them were not satisfied with their lives.

It’s hard to interpret the differences between different levels of life satisfaction in various communities, because several factors may have contributed to life satisfaction perception such as family values, economic status, and physical conditions and so on.

Therefore, a precise analysis cannot be proposed on this issue (Won & Choi, 2013).

The mean overall LSIA score was 22.1 ± 7.1 according to the present study. Subasi et al. reported the overall score to be 25.26 ± 5.51 (Subaşı & Hayran, 2005). In another study, Enkvist and Asa reported the overall score to be 23 (Enkvist, Ekström, & Elmståhl, 2012). In their study of 2005, Lowenstein et al. reported the mean life satisfaction score to be 20.3 ± 5.9 (Lowenstein & Katz, 2005). Franchignoni et al. reported the mean overall life satisfaction score to be 20.1 ± 6 (Franchignoni, Tesio, Ottonello, & Benevolo, 1999). Niknami et al. reported the mean score of life satisfaction in the elderly to be 20.19 ± 7.31 (Niknami et al., 2010). The mean score of life satisfaction obtained by the present study is not much different from the mean found by other studies. In fact, it is higher than the mean reported by another study conducted in Iran by Niknami et al. (Niknami et al., 2010). This finding seems somewhat reasonable as aging is normally accompanied by different changes in the body systems, reduced levels of activity and performance, as well as the emergence of diseases, which can all influence the overall satisfaction with life for the elderly.

In terms of the dimensions of the LSIA, the highest and lowest mean scores pertained to the resolution and fortitude (6.1 ± 2.5) and the positive self-concept (3.1 ± 1.2) subscales, in respective order. In a study conducted by Post et al., the highest and lowest mean scores pertained to the domain of companionship, friendship and communication (4.88) and the domain of leisure (3.80) (Post, Van Dijk, Van Asbeck, & Schrijvers, 1998).

In the study of Kimm et al., 281, or 34%, of the elderly people interviewed reported adequate life satisfaction (score 26-40), 316 people, or 38%, reported average life satisfaction (score 16-25) and 224 people, or 27%, reported poor life satisfaction (score 0-15) (Kimm, Sull, Gombojav, Yi, & Ohrr, 2012).

Considering the characteristics of old age, over time and as they find themselves faced with different situations, older individuals achieve a certain level of wisdom and patience that allow them to perceive problems more contemplatively than younger generations. The low mean score for the positive self-concept dimension might be due to the reality that their social environment and public atmosphere does not have a positive attitude toward the phenomenon of ageing. This, taken synonymously with disease and disability, is a perception that can affect the elderly population’s elderly self-concept.

Middle school and university level education were among the variables affecting the overall LSIA. A study conducted by Subaşı and Hayran confirms the level of education as a predictor of life satisfaction (Subaşı & Hayran, 2005). Kimm et al. (2012), revealed that average life satisfaction (score 16-25) and poor life satisfaction (score 0-15) was more common among participants with an elementary education level (Kimm et al., 2012).

They reported a higher degree of life satisfaction among married elderly that achieved the higher levels of education. Elderly study conducted by Light et al., also confirms the level of education as a factor influencing life satisfaction among this population. Enkvist et al. also reported a higher level of education to be an influential factor in determining the LSIA (Enkvist et al., 2012). In Kudo’s study, a higher level of education was closely linked to a higher life satisfaction and favorable health behaviors (Kudo et al., 2007).

Gana et al., (2013) found that the education was significantly related to life satisfaction in an eight-year cohort study. Increased education levels were significantly related to life satisfaction when using linear regression model (Gana, Bailly, Saada, Joulain, & Alaphilippe, 2013).

From this study it could be interpreted that a higher level of education provides aged individuals with better economic conditions, stronger social ties, a feeling of efficacy, and a more realistic outlook on life processes. These are all factors that can possibly affect life satisfaction among the elderly. The living arrangement variable (whether an individual lives with a spouse or alone) also influenced the overall LSIA. Subaşı and Hayran also confirmed marital status as a predictor of life satisfaction (Subaşı & Hayran, 2005).

Fernandez-Alonso’s study (2012) associated the lower score on the life satisfaction scale for older women to not having a stable partner (Fernández-Alonso et al., 2012). In fact, they reported a higher degree of life satisfaction among the married elderly. Enkvist et al. also found married elderly males had higher LSIA scores (Enkvist et al., 2012). In the research study of Won and Choi in 2013, having supportive resources like family, is known as an effective criterion of life satisfaction (Won & Choi, 2013). In the study of Li et al. in 2014, financial dependence on offspring was declared as an influential factor to the life satisfaction of elderly people (Li et al., 2014). According to a study conducted by Lee that examined factors influencing life satisfaction among the elderly living alone, individuals living alone experienced low levels of life satisfaction (Lee, 2004). One could interpret these findings by noting that family is a major source of support. Interpersonal relationships in families are effective in restraining stress and anxiety, and developing feelings of intimacy and safety, thus leading to positive effects on life satisfaction among the elderly. Consistent with the study conducted by Enkvist et al., the present study also found being male is a factor that positively affects the LSIA (Enkvist et al., 2012). Similarly, in the study of Ní Mhaoláin (2012), gender was considered an influential factor to life satisfaction among elderly people (Ní Mhaoláin et al., 2012).

In the study of Won and Choi in 2013, it was noted that men experience more life satisfaction (Won & Choi, 2013). This might be because men receive more support from their family and others compared to women. It might also allude to the cultural norms of Iran and the fact that, for the most part, Iranian men enjoy greater financial independence compared to women and is more publicly active. These arrangements make men less dependent on support from their families and others, and also make them more satisfied with the support they receive, if any. Therefore, men report higher levels of support received during old age than do women. Part of the variance of response (the score received for the LSIA dimensions), justified by the studied variables (R2) in the final regression model, varied between 0.09 for mood tone and 0.22 for positive self-concept.

In a study conducted by Pinquart and Sörensen, the social network was found to be the most significant factor influencing the LSIA among the elderly (Pinquart & Sörensen, 2000). In the study conducted by Arab et al., of all the demographic variables analyzed using the step-by-step logistic regression model, education level was found to be the only variable significantly affecting the elderly’s life satisfaction (Arab et al., 2011). Results of a study conducted by Gholizadeh and Shirani revealed a significant correlation between life satisfaction among the elderly and family factors (r=0.618), social factors (r=0.625), economic factors (r=0.656), personal independence (r=0.525) and physical health status (r=0.183) (Gholizadeh A & Shirani E, 2010). Results of a study conducted by Hosseini et al., showed that, among elderly women, there is a significant positive correlation between social support, life satisfaction and happiness, and a significant negative correlation between the aforementioned variables and depression (Hosseini, Rezaee, & Keykhosravi, 2012). Various studies have examined the correlation between numerous different factors and the LSIA among the elderly; consequently, different influential factors of LSIA among the elderly have been reported, making the interpretation the different findings difficult.

Limitations: Lack of a centralized location for having access to elderly individuals was one of the main constraints in this study. As such, the researcher visited multiple locations likely to have been visited by elderly individuals. Since many factors like economic status, mental disease, and financial problems affect the level of satisfaction of elder people, the researcher did not obtain information such as claims of depression, mental problems, or economic status, due to the subjective nature of these topics.

## 5. Conclusion

Given the findings of the present study and the mean overall LSIA score and its dimensions, we find that, in particular, positive self-concept as well as family and society’s perception of the phenomenon of aging should be revised. This primarily necessitates devising plans for reforming social attitudes toward the phenomenon of aging and for promoting, through different methods, respect for the elderly on a family and a societal level. We should be careful, however, not to allow support for the elderly to threaten their sense of independence, which can be avoided by consulting with the elderly themselves and receiving their own comments on the subject. In addition, further studies need to be done on elderly women, because more of the elderly are women and they face more problems with aging, specifically in traditional communities with limited participation in social activities and connections and need more support from their children and people nearby.
